# Synergistic metabolism of *Lactobacillus* and *yeast* at different inoculation improves the quality of fermented beef

**DOI:** 10.3389/fmicb.2026.1754736

**Published:** 2026-02-10

**Authors:** Xiaoxia Liu, Xiang Li, Zeyuan Lian, Pengjian Lv, Aiguo Luo

**Affiliations:** 1 Department of Biological Science and Technology, Shanxi Center of Technology Innovation for Compound Condiment, Jinzhong University, Jinzhong, China; 2 School of Food Science and Engineering, Shanxi Agricultural University, Jinzhong, China

**Keywords:** *Debaryomyces hansenii*, fermentation, flavor, *Lactobacillus plantarum*, metabolomics

## Abstract

**Background:**

Fermented meat products rely on complex microbial interactions to develop desirable safety, nutritional and sensory attributes. Lactic acid bacteria and yeasts are among the most frequently used starter cultures, yet their individual and interactive contributions to physicochemical changes and metabolite formation remain incompletely understood. In particular, how different inoculation ratios of bacteria and yeast regulate acidification, nitrogen metabolism and lipid remodeling in fermented meat has received limited attention. Therefore, this study investigated the effects of single and co-fermentation by *Lactobacillus plantarum* and *Debaryomyces hansenii* at different inoculation ratios on physicochemical properties and the non-volatile metabolome of fermented meat.

**Methods:**

Six fermentation treatments were designed, including an uninoculated control (A), *L. plantarum* alone (B), *D. hansenii* alone (C), and co-inoculated systems with lactic acid bacteria to yeast ratios of 1:1 (D), 1:2 yeast-dominant (E), and 2:1 lactic acid bacteria-dominant (F). After fermentation, pH, protein content and total volatile basic nitrogen (TVB-N) were determined to evaluate acidification, protein accumulation and nitrogen degradation. Untargeted non-volatile metabolomics was performed, and differential metabolites among treatments were screened using orthogonal partial least squares discriminant analysis (OPLS-DA).

**Results:**

Compared with the control (pH 6.01, protein 17.6 g per 100 g, TVB-N 8.4 mg per 100 g), *L. plantarum* fermentation markedly lowered pH to 4.72 and reduced TVB-N to 7.3 mg per 100 g. *D. hansenii* alone caused moderate acidification (pH 5.77), increased protein content to 21.9 g per 100 g, and resulted in a TVB-N level of 7.6 mg per 100 g. Co-fermentation showed clear ratio-dependent effects. Treatments D and F achieved lower pH values (4.55 and 4.45) with TVB-N levels of 6.2 and 5.6 mg per 100 g, respectively. The yeast-dominant treatment E exhibited the highest protein content (24.7 g per 100 g) and the lowest TVB-N (4.3 mg per 100 g). Metabolomics analysis identified 72 differential non-volatile metabolites, mainly lipids and nitrogen-containing compounds. Treatment E was characterized by increased free amino acids, phospholipids (e.g., LPC 18:2 and LPE 18:2), unsaturated fatty acids (linoleic and oleic acids), and nicotinic acid, whereas treatment F showed intensified phospholipid signals (e.g., PC 34:1) accompanied by depletion of multiple amino acids and nicotinamide.

**Discussion:**

The results demonstrate that the inoculation ratio of *L. plantarum* and *D. hansenii* plays a decisive role in regulating acidification, proteolysis and lipid remodeling during meat fermentation. Yeast-dominant co-fermentation promoted protein accumulation and enrichment of unsaturated fatty acids, likely through enhanced proteolytic activity and lipid metabolism, while effectively limiting TVB-N formation. In contrast, lactic acid bacteria-dominant fermentation intensified acidification and further suppressed nitrogenous spoilage indicators, albeit with reduced amino acid availability. These findings highlight the importance of microbial balance in starter culture design and provide a mechanistic basis for tailoring fermentation strategies to simultaneously improve safety and sensory quality of fermented meat products.

## 1 Introduction

Amid tightening regulatory scrutiny and growing industry interest in refining the sensory quality of traditional fermented meat products, the directed use of selective starter cultures to steer microbial succession, reprogram fermentative metabolic fluxes, and enhance sensory properties and storage stability has emerged as a key strategy for product improvement (Wang et al., 2021, Marta et al., 2019, Van Reckem et al., 2019, Li et al., 2019). *Lactobacillus plantarum* is widely employed in meat fermentations because of its strong acidification capacity, broad environmental adaptability and diverse enzymatic repertoire (including both extracellular and intracellular systems). These traits enable rapid pH decline, suppression of spoilage and pathogenic microorganisms, and modulation of protein, peptide and free amino acid profiles-thereby altering the pool of flavor precursors and improving antioxidative and anti-spoilage properties (Van Wyk et al., 2023, Miao et al., 2024, Chen et al., 2024, Yang et al., 2022). Complementing this role, *non-Saccharomyces* yeasts such as *Debaryomyces hansenii* have attracted attention across fermentative systems for their rich extracellular enzyme activities (*β*-glucosidase, proteases, esterases, etc.) and their capacity to liberate glycosylated aroma precursors and to catalyze protein hydrolysis. These functions have been shown to amplify aromatic complexity and reshape small-molecule metabolism, suggesting that such yeasts can act as valuable co-inoculants for diversifying flavor and improving texture (Gong et al., 2024, Windarsih et al., 2024, Sidira et al., 2024). Recent studies indicate that mixed inoculation of *lactic acid bacteria* and *non-Saccharomyces* yeasts frequently produces synergistic effects: the rapid acidification and antagonistic interactions driven by *lactic acid bacteria* limit accumulation of volatile basic nitrogenous compounds, while yeast-mediated enzymatic transformations expand the pools of soluble peptides, free amino acids and volatile aroma precursors. The metabolic complementarity of these organisms at the network level therefore often achieves concurrent gains in flavor complexity and product safety/stability (Zhang et al., 2025, Zhou et al., 2022, Hua et al., 2022). To systematically uncover how microbial interactions under different inoculation ratios reshape metabolic networks and drive flavor formation, non-targeted liquid chromatography-high-resolution mass spectrometry (LC-HRMS/LC–MS) metabolomics combined with multivariate statistics (PCA, OPLS-DA), hierarchical clustering, Venn analysis and KEGG pathway enrichment has become a standardized methodological framework for dissecting reconfigurations of amino acid, peptide, lipid and nucleotide metabolism and their associations with sensory and safety indices (Alabi et al., 2025).

Building on this background, the present study employs non-targeted LC–MS metabolomics to compare single inoculations and mixed inoculations of *L. plantarum* and *D. hansenii* during raw beef fermentation, with the aim of providing molecular-level evidence and practical guidance for starter-ratio design that co-optimizes flavor and safety in fermented meat products.

## 2 Materials and methods

### 2.1 Experimental materials and instruments

Pingyao beef was purchased from Shanxi Pingyao Beef Group Co., Ltd. (Pingyao, Shanxi, China). *Lactiplantibacillus plantarum* and *Debaryomyces hansenii* were obtained from the Shanghai Culture Collection Center (Shanghai, China). Analytical-grade inorganic salts and organic reagents used in this study included: dipotassium hydrogen phosphate (K_2_HPO_4_), sodium acetate, glucose, Tween 80, manganese sulfate, agar, magnesium sulfate, sodium hydroxide, yeast extract, calcium carbonate, phenolphthalein, diammonium citrate, and anhydrous ethanol (all purchased from Tianjin Beichen Fangzheng Reagent Factory, Tianjin, China). The following instruments were used: Vanquish ultra-high performance liquid chromatograph (UHPLC) and Q-Exactive HF high-resolution mass spectrometer (Q-Exactive HF) (Thermo Fisher Scientific, Waltham, MA, United States); TGL-16 M centrifuge (Xiangyi, Changsha, Hunan, China); JXFSTPRP-48 automatic sample rapid grinder (Jingxin Instrument Co., Ltd., Shanghai, China); KQ-00DE CNC ultrasonic cleaner (Kunshan Ultrasonic Instruments Co., Ltd., Kunshan, Jiangsu, China); FD-IA-50 freeze dryer (Shanghai Bilang Instrument Manufacturing Co., Ltd., Shanghai, China); AUW-120D balance (Shimadzu Corporation, Kyoto, Japan); GWB-2B ultrapure water system (Puxi Analytical Instruments Co., Ltd., Beijing, China); and G560E vortex mixer (Scientific Industries, Inc., New York, NY, United States).

### 2.2 Experimental group design

All samples were taken from the same batch of raw beef and were subjected to different starter combinations as follows Table 1.

**Table 1:** Experimental design.

Group	Vaccination schedule
A	Not vaccinated
B	Only vaccinated with *Lactobacillus plantarum*
C	Only vaccinated with *Debaryomyces hansenii*
D	*Lactobacillus plantarum*: D*ebaryomyces hansenii* = 1:1
E	*Lactobacillus plantarum*: D*ebaryomyces hansenii* = 1:2
F	*Lactobacillus plantarum*: D*ebaryomyces hansenii* = 2:1

### 2.3 Basic fermentation conditions

All fermentations were carried out under controlled and uniform conditions to ensure experimental consistency: the incubation temperature was maintained at 25 °C for 16 h, with a relative humidity of 60%. The total inoculation level of starter cultures was 1.5% (w/w), calculated based on the weight of the fresh beef. For each treatment, the bacterial and yeast suspensions were adjusted to a concentration of 1 × 10^8^ CFU/mL prior to inoculation.

### 2.4 Sample preparation and extraction

After deodorisation with cooking wine, the beef was divided into groups according to the inoculation treatments for fermentation. Following fermentation, the samples were subjected to a standardized cooking procedure to simulate practical processing conditions. Briefly, cooking oil was preheated until shimmering, then 5 g of scallion, 3 g of ginger, and 5 g of garlic were added and stir-fried until aromatic. The fermented beef was subsequently added and stir-fried together, followed by the addition of 5 g of chili pepper, 2 g of Sichuan peppercorns, and 2 g of star anise. After 3 min of stir-frying, salt was added for seasoning. The cooked samples were then packed into sterilized jars and subjected to water-bath sterilization, yielding the final products for analysis.

For metabolite extraction, an accurately weighed portion of each homogenized sample was transferred into a 2 mL centrifuge tube. 1 mL of 70% methanol and a 3 mm stainless-steel bead were added, and the mixture was homogenized for 3 min using an automatic grinder. The samples were then vortexed for 10 min to ensure complete extraction. After centrifugation at 12,000 rpm for 10 min at 4 °C, the supernatant was collected and filtered through a 0.22 μm microporous membrane filter for subsequent LC–MS analysis.

### 2.5 Chromatography-mass spectrometry analysis

Liquid chromatography (LC) separation was performed using a Zorbax Eclipse C18 column (1.8 μm, 2.1 mm × 100 mm) maintained at 30 °C. The mobile phase consisted of 0.1% formic acid in water (A) and acetonitrile (B), delivered at a flow rate of 0.3 mL min^−1^ with an injection volume of 2 μL. The autosampler temperature was held at 4 °C. A linear gradient elution was applied as follows: starting with 5% B (95% A), the proportion of acetonitrile was gradually increased, returning to the initial condition at 21 min to re-equilibrate the column.

Mass spectrometric detection was conducted in both positive and negative electrospray ionization (ESI) modes with a spray voltage of 3.5 kV. The capillary temperature was 330 °C, the heater temperature 325 °C, and the S-Lens RF level was set to 55%. The sheath, auxiliary, and sweep gas flow rates were 45, 15, and 1 arb units, respectively. Data were acquired in full-scan mode (m/z 100–1,500) and data-dependent MS/MS (Top *N* = 5) using higher-energy collisional dissociation (HCD). The mass resolution was 120,000 for MS^1^ scans and 60,000 for MS^2^ scans.

### 2.6 Detection of physical and chemical indicators

Protein content was determined according to the Chinese National Standard GB 5009.5–2016, volatile basic nitrogen (TVB-N) was measured following GB 5009.228–2016, and pH was assessed in accordance with GB 5009.237–2016.

### 2.7 Data analysis

Chromatography-mass spectrometry (LC–MS) analysis was performed on the extracted samples, and data quality was evaluated by overlapping the total ion chromatograms (TICs) of quality control (QC) samples. Raw MS data were processed for retention time correction, peak detection, extraction, integration, and alignment, resulting in a comprehensive metabolite data matrix. Metabolite identification was achieved through spectral matching against the Thermo mzCloud, mzVault, and ChemSpider databases. The normalized data matrix was then subjected to multivariate statistical analyses, including principal component analysis (PCA) and orthogonal partial least squares discriminant analysis (OPLS-DA), in combination with univariate statistical testing (Student’s t-test) to identify differential metabolites. Differential features were further interpreted through hierarchical clustering heatmap analysis, KEGG pathway annotation, and pathway enrichment analysis to elucidate their biological significance.

## 3 Results

### 3.1 Effects of *Lactobacillus plantarum* and *Debaryomyces hansenii* yeast on pH, TVB-N, and protein in fermented meat products: single and co-fermentation

**Figure 1:**
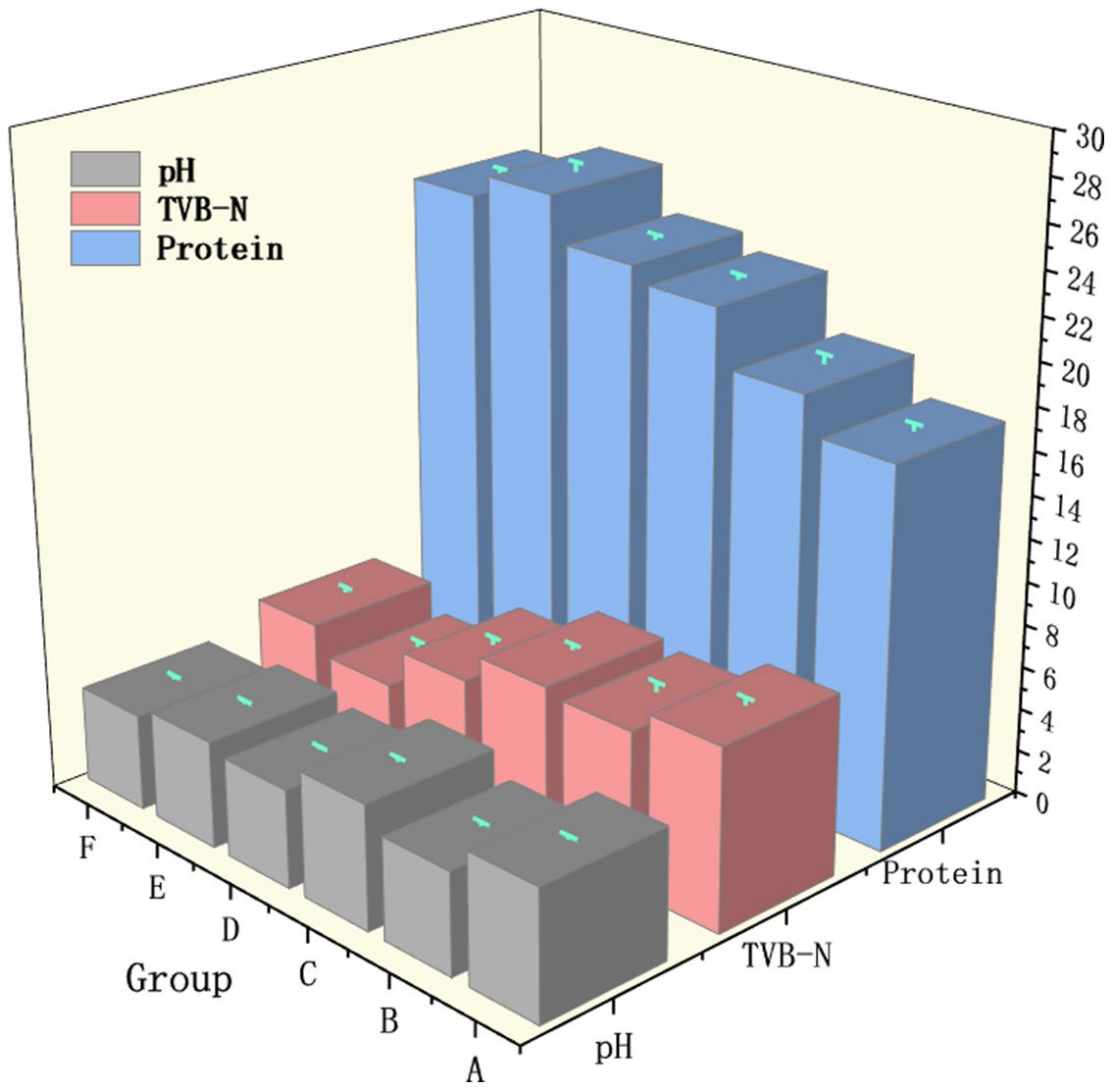
pH, TVB-N, and protein of the non-fermented group and different bacterial fermentation groups.

As shown in Figure 1, the control group A maintained a relatively high pH of 6.01, together with low measured protein content at 17.6 g/100 g and high TVB-N at 8.4 mg/100 g, indicating that the unfermented samples largely retained the characteristics of raw meat. In group B, inoculation with *Lactobacillus plantarum* alone led to pronounced acidification, with pH decreasing to 4.72, accompanied by a reduction in TVB-N to 7.3 mg/100 g. In contrast, fermentation with *Debaryomyces hansenii* alone in group C resulted in only a moderate pH decrease to 5.77 but a marked increase in protein content to 21.9 g/100 g, while TVB-N declined slightly to 7.6 mg/100 g. Mixed cultures in groups D, E, and F exhibited clear synergistic effects. Co-inoculation at a 1:1 ratio in group D or a *lactic acid bacteria*-dominant 2:1 ratio in group F produced stronger acidification, with pH values of 4.55 and 4.45, respectively, and further reductions in TVB-N to 6.2 and 5.6 mg/100 g. By contrast, the *yeast*-dominant 1:2 ratio in group E yielded the highest protein content at 24.7 g/100 g and the lowest TVB-N at 4.3 mg/100 g.

### 3.2 Effects of single and co-fermentation of *Lactobacillus plantarum* and *Debaryomyces hansenii* on the metabolomics of fermented meat products

**Figure 2:**
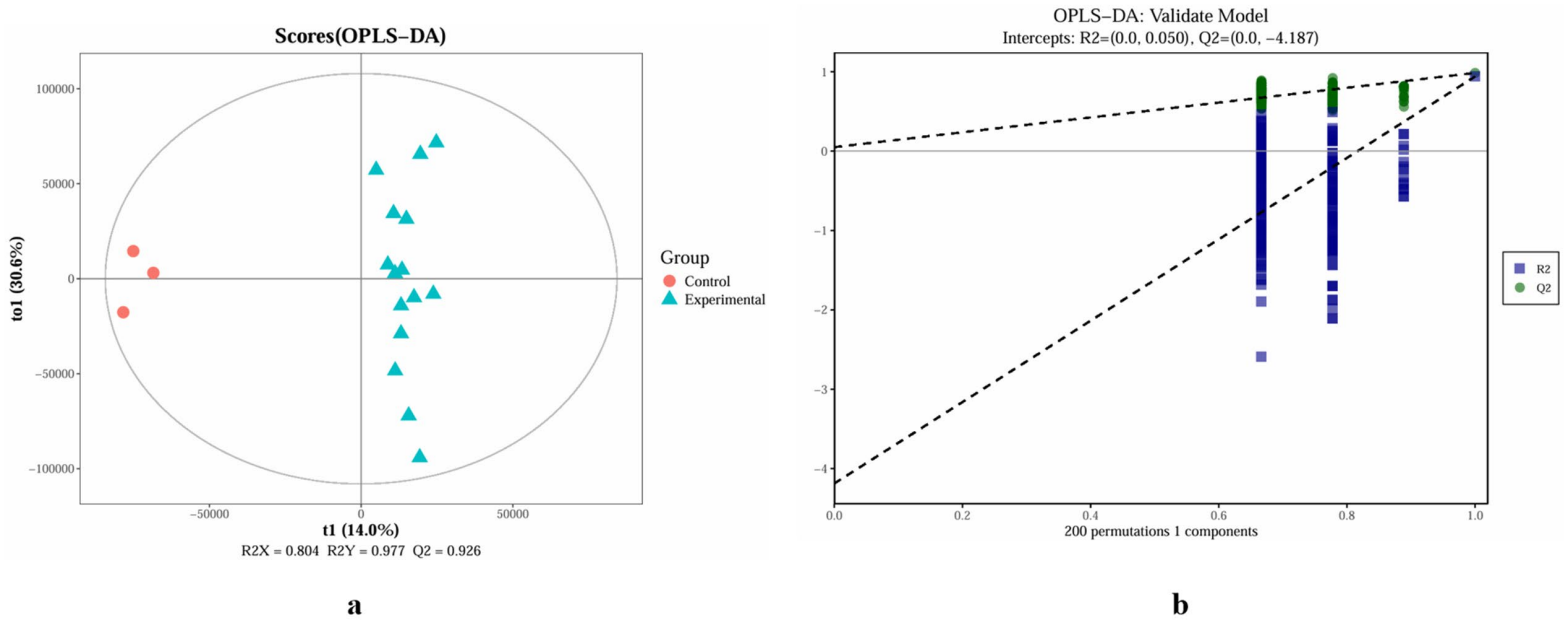
Partial least squares discriminant analysis (OPLS-DA) plot of non-volatile metabolite profiles in non-fermented and different strain-fermented groups **(a)** and model validation of the OPLS-DA model **(b)**.

Partial least squares-discriminant analysis (OPLS-DA) was performed to compare the non-volatile profiles of the control and experimental groups (Figure 2a). The OPLS-DA score plot illustrates the degree of clustering and dispersion among samples: points that are close together represent samples with more similar compositions and concentrations of the measured variables/metabolites, whereas points that are farther apart indicate greater differences between samples. The clear separation observed between the experimental groups and the control indicates effective class discrimination. An orthogonal partial least squares-discriminant analysis (OPLS-DA) model was subsequently constructed and showed high explanatory and predictive metrics (*R*^2^Y = 0.977, Q^2^ = 0.926; Figure 2b), indicating good model fit and predictive ability. Overall, the addition of starter strains and the fermentation process induced pronounced changes in the metabolic profiles of the experimental groups relative to the control.

### 3.3 Changes in differential metabolites during single and co-fermentation of *Lactobacillus plantarum* and *Debaryomyces hansenii*

**Figure 3:**
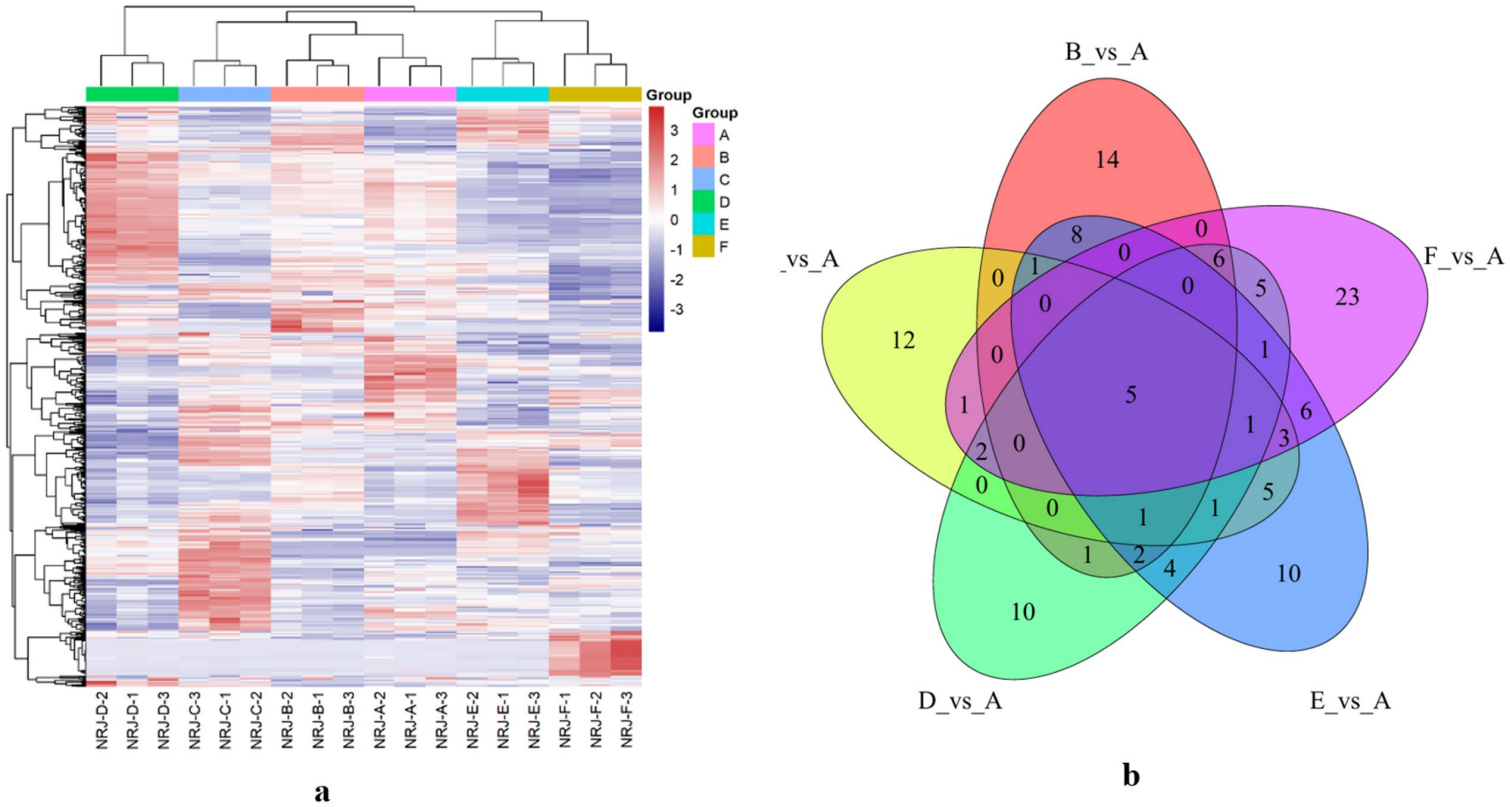
Heatmap **(a)** and Venn diagram **(b)** of non-volatile metabolite profiles in the non-fermented group and groups fermented with different strains.

Figure 3 shows that group B exhibited a slightly higher mean peak area than the control A, reaching 9.40 × 10^6^ compared with 8.12 × 10^6^, together with an approximately 1.8-fold enrichment of amino acids and their derivatives. In group C, the mean peak area increased to 9.35 × 10^6^, representing about a 15% rise relative to A, and increased further in group D to 9.76 × 10^6^. Lipid metabolism remained highly active in group E, with a mean peak area of 1.34 × 10^7^, corresponding to roughly 1.65-fold that of the control. Despite stronger acidification in group F, this treatment produced the most intense phospholipid signal, with a mean peak area of 1.73 × 10^7^, approximately 2.13-fold higher than A. Consensus clustering identified 72 differential metabolites with absolute Z-scores of at least 2, among which PC 34:1, LPE 18:2, acetylcarnitine, and branched-chain amino acids were prominent. Notably, LPE 18:2 increased by about 2.5-fold in group C but decreased by approximately 45% in group F. Most differential features were lipids and nitrogen-containing compounds, which together accounted for around 73.5% of the metabolic changes. Pairwise comparisons with the control yielded 14, 12, 10, 10, and 23 unique metabolites in groups B, C, D, E, and F, respectively, while five metabolites were shared across all comparisons, including NA, NAM, LysoPAF C18, AP, and AHMB. These common metabolites point to coordinated regulation of niacin and nicotinamide metabolism, phospholipid turnover, and aromatic compound transformation. Based on these distinctions, subsequent analyses focused on groups E and F, using group A as the control, to further evaluate how inoculation ratio governs lipid metabolism and amino-acid conversion.

#### 3.3.1 Changes in differential metabolites during fermentation in groups E and F

**Figure 4:**
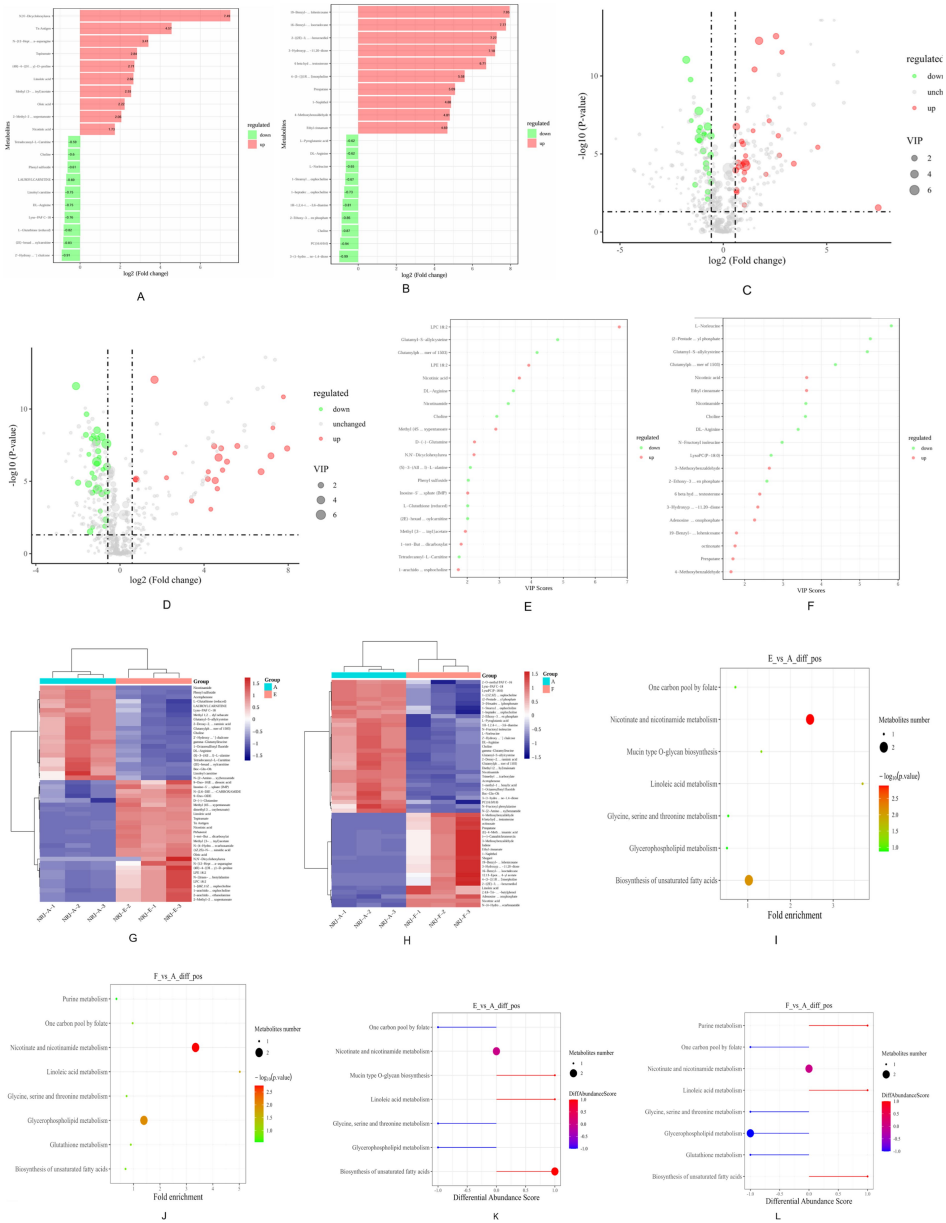
Bar chart of fold differences between pairs E and A, F and A **(A,B)**, volcano plot of differential metabolites **(C,D)**, VIP scores plot **(E,F)**, clustering heatmap of differential metabolites **(G,H)**, KEGG enrichment bubble chart **(I,J)**, differential abundance score plot **(K,L)**.

Figure 4 shows distinct metabolic responses in groups E and F relative to the control. Group E contained 27 upregulated metabolites, dominated by amino acids, purines, phospholipids, organic acids, and fatty acids. Among amino acids, M-Boc-AHP increased with a log_2_ fold change of 0.63 and a high VIP value, while D-Gln showed a pronounced rise. The purine metabolite IMP was also elevated, indicating enhanced nucleotide turnover. Phospholipids, particularly LPC 18:2 and LPE 18:2, were markedly increased, together with organic acids such as NA and M-BIA. Unsaturated fatty acids, including oleic acid and linoleic acid, showed strong accumulation. In addition, several biogenic amines were upregulated, with DCU exhibiting the largest increase. By contrast, 21 metabolites were reduced in group E, notably dipeptides such as Glu-SAC and Glu-Phe, the purine-related compound NAM, multiple acylcarnitines, and choline, indicating active nitrogen turnover and phospholipid remodeling. Overall, the metabolic profile of group E was characterized by a clear predominance of upregulated features.

Group F displayed a different pattern, with 22 metabolites upregulated, mainly fatty acids, organic acids, and biogenic amines. NA increased significantly, while several lipid-related compounds, including EC, OCT, and 4-MBA, showed sharp elevations. Biogenic amines such as HPD and 6β-OH-T also increased markedly, reflecting intensified secondary metabolism. In contrast, 31 metabolites were downregulated in group F, largely comprising amino acids and phospholipids. These included NLE, Glu-SAC, Glu-Phe, Arg, and Fru-Ile, together with a pronounced decrease in NAM. Phospholipid-related metabolites such as PDMEP, choline, and LysoPC P-18:0 were also reduced. In addition, several organic acids and small molecules showed lower levels.

#### 3.3.2 Analysis of metabolic pathways during fermentation in group E

**Figure 5:**
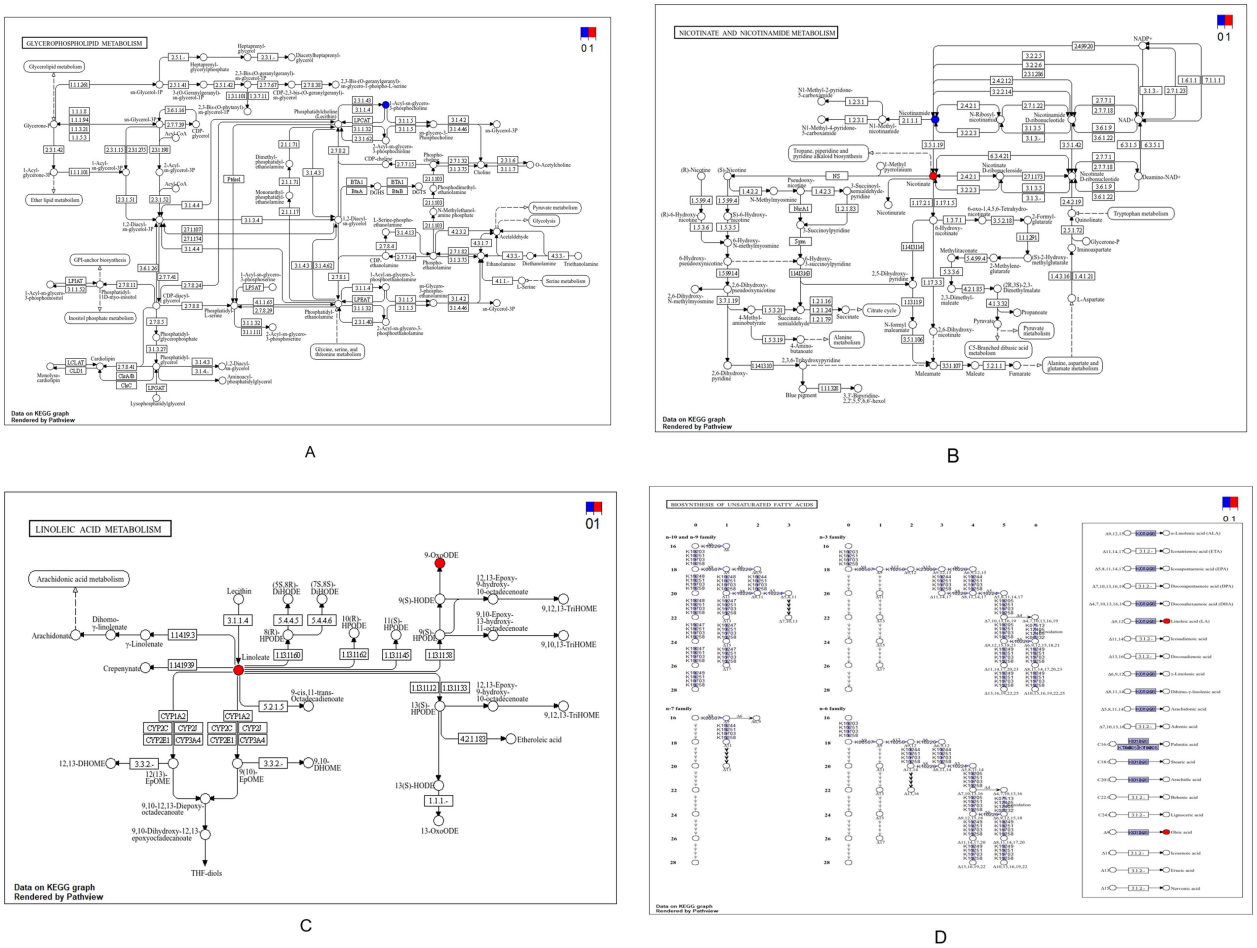
Differential metabolic pathway map of the EvsA group. **(A)** Glycerophospholipidmetabolism; **(B)** Metabolismofniacinandnicotinamide; **(C)** Linoleicacidmetabolism; **(D)** Biosynthesisofunsaturatedfattyacids.

Figure 5 shows a pronounced decrease in choline in group E. The biosynthetic sequence from phosphocholine to CDP-choline and further to phosphatidylcholine was significantly enriched, with key enzymes K06121, K00993, and K00994 prominently involved. In the nicotinate and nicotinamide metabolism pathway, nicotinic acid was significantly upregulated, whereas nicotinamide was reduced. The linoleic acid metabolism pathway displayed elevated levels of linoleic acid and its oxidative derivatives, including 9-oxo-ODE and other HODE and HPODE products. In parallel, the biosynthesis of unsaturated fatty acids was also enriched, with both linoleic acid and oleic acid showing significant increases in group E.

## 4 Discussion

Microbial co-fermentation is increasingly recognized as a powerful strategy to shape flavor development and nutritional quality in fermented meat products. The benefits observed in the present study arise from metabolic complementarity rather than from simple additive effects. Specifically, coordinated substrate release, metabolic rerouting, and redox balancing between *Lactobacillus plantarum* and *Debaryomyces hansenii* explain the improved outcomes (Ba et al., 2018; Sidira et al., 2024).

A central feature of *yeast*-*lactic acid bacteria* co-fermentation is the division of metabolic labor. *Yeast* contribute primarily to macromolecule degradation and substrate mobilization. *Lactic acid bacteria* excel at rapid carbohydrate consumption, acidification, and directing metabolism through redox-linked pathways (Liu et al., 2023; Sidari and Tofalo, 2024). This functional asymmetry explains the greater amino-acid availability observed under mixed-culture conditions. Rather than simply increasing proteolysis, *yeast*-mediated peptide release expands the accessible nitrogen pool. *Lactic acid bacteria* then convert this pool into flavor-active amino acids and downstream metabolites. Similar synergistic nitrogen dynamics have been reported in fermented sausages and in dairy matrices, where *yeast* activity raises amino-acid flux without compromising microbial stability (Leroy et al., 2005; Flores, 2018).

Lipid remodeling emerges as a defining process that links cellular adaptation to aroma precursor formation during co-fermentation. Glycerophospholipid turnover now appears to act as a metabolic interface between membrane adaptation and aroma generation (Cao et al., 2023; Yang et al., 2024). In mixed cultures, intensified phospholipid hydrolysis likely reflects membrane restructuring under acidic and metabolically demanding conditions. This remodeling helps preserve cell integrity and permeability. It also releases lysophospholipids and free fatty acids that serve as substrates for subsequent oxidative reactions (Fan et al., 2025; Indriani et al., 2024). Such membrane-driven substrate release helps explain why mixed cultures consistently increase lipid-derived flavor potential relative to mono-culture systems.

Redox metabolism coordinates these transformations. The nicotinate and nicotinamide pathway functions as a metabolic hub that integrates energy production, oxidative capacity, and biosynthetic demand. Enhanced nicotinamide adenine dinucleotide turnover has been associated with intensified lipid oxidation and increased dehydrogenase activity in fermented foods (Migaud et al., 2024; Mei et al., 2024). In co-fermentation systems, *yeast* metabolites together with *lactic acid bacteria*-driven acidification shift the intracellular redox state. This shift favors nicotinamide adenine dinucleotide-dependent reactions. The resulting redox reprogramming sustains fatty-acid oxidation and amino-acid transformation, thereby coupling energy metabolism to the synthesis of flavor precursors (Zhang et al., 2021).

Linoleic acid metabolism is a downstream convergence point for these processes. Unsaturated fatty acids, especially linoleic acid, are well established as precursors of aldehydes, ketones, and alcohols that shape the sensory character of fermented meat products (Dragoev, 2024). The greater availability of linoleic acid during co-fermentation comes not only from increased lipid hydrolysis but also from preferential retention and accumulation of unsaturated species. These changes improve membrane fluidity and increase enzyme accessibility. Subsequent enzymatic or non-enzymatic oxidation of these fatty acids yields oxylipins. Oxylipins act both as aroma precursors and as signaling molecules linked to oxidative stress responses. This dual role highlights the tight coupling between cellular adaptation and flavor formation in mixed microbial ecosystems (Chen et al., 2025).

Microbial dominance within mixed cultures determines whether metabolic fluxes favor precursor accumulation or direct formation of volatile aroma compounds. When *yeast* activity is relatively strong, metabolic networks bias toward substrate release and redox preparation, creating a reservoir of nutritional and flavor precursors. Conversely, when *lactic acid bacteria* predominate, pathways tend to advance lipid oxidation and volatile compound biosynthesis, which accelerates the accumulation of low-molecular-weight aroma compounds that determine sensory perception (Liu et al., 2025). This balance mirrors observations in other fermented food systems, where community composition governs the shift from primary metabolism to secondary aroma production (Li et al., 2025; Smid and Lacroix, 2013; McSweeney and Sousa, 2000).

From an applied perspective, these results reinforce the view that inoculation ratio is more than a processing parameter: it is a metabolic control lever. By tuning interactions between *yeast* and *lactic acid bacteria*, practitioners can steer fermentation toward improved nutritional value, greater aroma complexity, or targeted flavor profiles. This approach aligns with strategies in directed fermentation and in microbial consortia engineering that prioritize metabolic predictability over single-strain robustness. Understanding the metabolic logic of co-fermentation therefore provides a rational basis for starter culture design and for targeted flavor modulation in fermented meat products (Wang et al., 2025; Liu et al., 2025).

## 5 Conclusion

Co-fermentation of *Lactobacillus plantarum* and *Debaryomyces hansenii* is primarily governed by the inoculation ratio, which significantly regulates acidification, protein degradation, and lipid remodeling in fermented meat. *Yeast*-dominated inoculation promotes protein accumulation and enriches unsaturated fatty acids and phospholipids, whereas *lactic acid bacteria*-dominated inoculation accelerates acidification and reduces total volatile basic nitrogen, thereby contributing to effective microbial quality control. The inoculation ratio thus represents a critical lever for modulating both the safety and sensory attributes of fermented meat products. These findings provide practical guidance for starter culture selection and process optimization in fermented meat production.

## Data Availability

The original contributions presented in the study are included in the article/supplementary material, further inquiries can be directed to the corresponding author/s.
